# Real-Time Continuous Monitoring of Oral Soft Tissue Pressure with a Wireless Mouthguard Device for Assessing Tongue Thrusting Habits

**DOI:** 10.3390/s23115027

**Published:** 2023-05-24

**Authors:** Hidekazu Matsumoto, Keisuke Tomoto, Gentaro Kawase, Kenta Iitani, Koji Toma, Takahiro Arakawa, Kohji Mitsubayashi, Keiji Moriyama

**Affiliations:** 1 Department of Maxillofacial Orthognathics, Graduate School of Medical and Dental Sciences, Tokyo Medical and Dental University, Tokyo 113-8549, Japank-moriyama.mort@tmd.ac.jp (K.M.); 2 Department of Biomedical Devices and Instrumentation, Institute of Biomaterials and Bioengineering, Tokyo Medical and Dental University, Tokyo 101-0062, Japan; 3 Department of Electronic Engineering, Shibaura Institute of Technology, College of Engineering, Tokyo 135-8548, Japan; 4 Department of Electric and Electronic Engineering, Tokyo University of Technology, Tokyo 192-0982, Japan

**Keywords:** pressure sensor, wireless, mouthguard, oral soft tissue pressure, tongue thrust

## Abstract

In orthodontics, understanding the pressure of oral soft tissues on teeth is important to elucidate the cause and establish treatment methods. We developed a small wireless mouthguard (MG)-type device that continuously and unrestrainedly measures pressure, which had previously been unachieved, and evaluated its feasibility in human subjects. First, the optimal device components were considered. Next, the devices were compared with wired-type systems. Subsequently, the devices were fabricated for human testing to measure tongue pressure during swallowing. The highest sensitivity (51–510 g/cm^2^) with minimum error (CV < 5%) was obtained using an MG device with polyethylene terephthalate glycol and ethylene vinyl acetate for the lower and upper layers, respectively, and with a 4 mm PMMA plate. A high correlation coefficient (0.969) was observed between the wired and wireless devices. In the measurements of tongue pressure on teeth during swallowing, 132.14 ± 21.37 g/cm^2^ for normal and 201.17 ± 38.12 g/cm^2^ for simulated tongue thrust were found to be significantly different using a *t*-test (n = 50, *p* = 6.2 × 10^−19^), which is consistent with the results of a previous study. This device can contribute to assessing tongue thrusting habits. In the future, this device is expected to measure changes in the pressure exerted on teeth during daily life.

## 1. Introduction

Sensor devices are widely used in clinical practice to measure physical parameters that serve as indicators of various human health conditions [1,2,3,4]. Currently, physical parameters are measured by medical professionals at medical institutions within a limited time period. However, since physical parameters are affected by circadian rhythms and body position, they should be monitored continuously throughout the day to understand diurnal fluctuations and time series data. Thus, in recent years, several studies have been conducted on the research and development of wearable (bio)sensors that enable continuous measurement [5].

For instance, we developed an intraoral mouthguard (MG)-type biosensor for the continuous monitoring of the salivary glucose concentration, which reflects blood glucose levels [6]. These devices would be beneficial for the simple monitoring of blood glucose fluctuations caused by eating, sleeping, and taking medications. In addition, a contact lens-type pressure sensor was developed to measure intraocular pressure, which is the greatest risk factor for glaucoma in preventing blindness [7].

**Figure 1:**
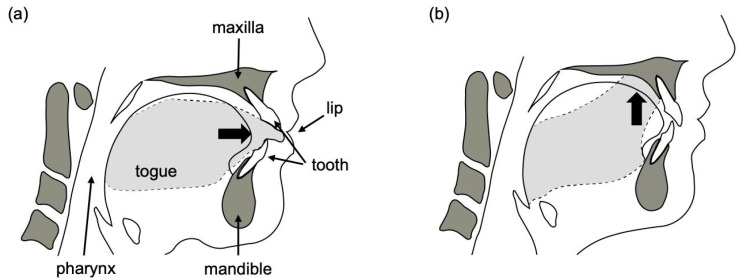
(**a**) Tongue thrust type of swallowing; (**b**) mature type of swallowing.

Measurements of several useful physical parameters can be acquired from the oral cavity, which has various functions, such as eating, swallowing, breathing, and speech. In orthodontics, the “equilibrium theory” is widely accepted. This theory explains that the perioral muscles influence the position of the teeth and the dental arch morphology. Therefore, the pressure exerted on teeth by the tongue and perioral muscles has long attracted attention. Previously, it was reported that an imbalance among the pressure from the lips, buccal mucosa, and tongue induces malocclusion [8,9,10,11]. Tongue thrust is an oral habit that affects the balance of oral soft tissue pressure. Tulley defined tongue thrust as the forward movement of the tongue tip between the upper and lower teeth, reaching the lower lip during deglutition and speech (see Figure 1) [12]. Although it varies from report to report, tongue thrust is generally considered to potentially be associated with increased overjet, open bite, proclined maxillary anterior teeth, small maxillary dental arch width, increased upper lip thickness, mouth breathing, and lip incompetency [13,14,15,16].

Currently, several studies have been conducted on the measurement of pressure on teeth from oral soft tissue pressure using sensor devices to elucidate the causes of malocclusions and establish treatment methods [17,18,19,20,21,22,23,24]. However, to the best of our knowledge, previous studies have not measured pressure inside the oral cavity under natural conditions. Most studies used pressure sensors that were wired to a power supply and a recording device installed outside the oral cavity. A few wireless communication devices have also been reported, but the components in the mouth were large [25]. The abovementioned methods have a few drawbacks. First, electrical wires and other components of a device may affect the movement and measurement results of oral soft tissue pressure. Second, wired devices require a power supply and connection to peripheral equipment, so measurements must be taken under certain conditions, such as body position. The pressure of oral soft tissue changes not only during breathing, chewing, and speaking but also in response to changes in the body and head position. Therefore, it is expected to change in a complex manner during daily activities [26,27,28,29,30]. In addition, since it is known that physiological conditions, such as respiratory dynamics and muscle tone, differ between the sleep and awake periods [31], measurements taken only under specific conditions are considered insufficient to ascertain oral soft tissue pressure on the teeth. Hence, we wanted to develop a sensor device that would solve both of these problems and enable the measurement of the complex diurnal variation in oral soft tissue pressure on the teeth.

The aim of this study was to develop an intraoral sensor device that solves these problems and enables real-time continuous measurement of oral soft tissue pressure without restraints. The feasibility of the developed device was evaluated in a study with human.

## 2. Materials and Methods

**Figure 2:**
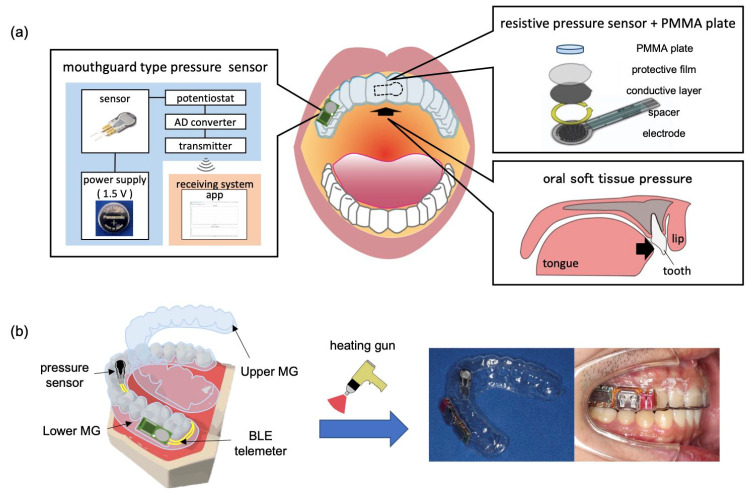
(**a**) Schematic diagram of the MG-type pressure sensor device; (**b**) fabrication method and overview of the MG-type pressure sensor devices.

The components of the oral sensor device include a pressure sensor, small circuit to acquire data, data transmitter to realize unrestrained measurement, and battery to power these components. When placed in the oral cavity, it is necessary to prevent inflammation of the oral cavity, which may occur due to the presence of these components. Therefore, all components were packed with mouthguard material to ensure safety. A schematic of the device is shown in Figure 2a. The method fabrication and overview of the device are shown in Figure 2b.

The pressure sensor used in this system should have a dynamic range of 58.9–154.12 g/cm^2^ [22,32,33], which is the pressure range reported so far for the lateral surface of the maxillary anterior palate during swallowing. In addition, it must not be affected by temperature changes and should not cause discomfort when placed inside the mouth. Therefore, a resistive pressure sensor [17,34] with a sufficiently thin thickness was employed (FSR400 short, upper right of Figure 2a; measurable range: 102–10,710 g/cm^2^, width: 6.35 mm, height: 15.8 ± 0.15 mm, thickness: 0.3 ± 0.03 mm, and size of sensing area: diameter of 5.08 mm, Interlink Electronics, Camarillo, CA, USA).

### 2.1. Examination of Sensors Suitable for Measuring Oral Soft Tissue Pressure

#### 2.1.1. Selection of a Pressure Transmission Element

When the pressure sensor is packed with MG material, the pressure is absorbed by the MG material on the upper and lower surfaces of the pressure sensor, and the pressure is not accurately transmitted to the sensor. Therefore, we investigated a method to improve the transmission efficiency of oral soft tissue pressure to the pressure sensor by placing a circular and high modulus material on the sensitive area of the pressure sensor. In this study, a plate of polymethyl methacrylate (PMMA) was selected because it is not deformed by oral soft tissue and is easy to mold. The PMMA plate was visually and manually positioned so that the center of the sensor’s sensitive area was aligned with the center of the plate.

The response of the pressure sensor varied depending on the size of the circular PMMA plate (thickness: 1.0 mm) used in this method. The suitable diameters of the PMMA plates for pressure transmission were selected as 2, 3, 4, and 5 mm. In the fabrication of the experimental samples, the pressure sensor was placed on the MG material (Erkoflex, ERKODENT, Pfalzgrafenweiler, Germany) made of ethylene vinyl acetate (EVA) on a flat surface, and each circular PMMA plate was placed at the center of the sensitive area of the pressure sensor. Subsequently, the MG material made of EVA was heated and softened in a vacuum-forming machine (Vacuum Adapter I, Yamahachi Dental, Aichi, Japan) and pressed from above (see Figure S1). To ensure that all of the MG materials were of equal thickness, the vacuum-forming matinee was viewed from the side during heating, and the MG materials were pressed when they were hanging down approximately 1.5 mm. To evaluate the characteristics of the pressure sensor, various pressures were applied to the experimental samples using a tensile and compression testing machine (SV-55C, Imada Seisakusho, Aichi, Japan). The current generated by the change in the resistance of the pressure sensor was measured by applying a constant potential of +400 mV using a potentiostat (Model 1112, Husou Electrochemical System, Kanagawa, Japan), and the pressure transfer efficiency was compared.

#### 2.1.2. Selection of MG Material for 2D Packaging

The composition of the MG material is considered to affect the response to the pressure of the packaged sensor. The MG material used for the upper and lower surfaces of the pressure sensor was made of EVA (thickness: 1.0 mm and modulus: 7.1 × 10^5^ g/cm^2^) and polyethylene terephthalate glycol (PETG) (thickness: 0.5 mm and modulus: 2.1 × 10^7^ g/cm^2^). Each of the four combinations was then compared. Each examination was conducted five times and evaluated.

#### 2.1.3. Selection of MG Materials for 3D Packaging

The three-dimensional formation of the MG was performed by pressing the heat-softened MG material against the plaster dental model. Because the plaster dental model has a complex morphology, the thickness of the MG after its formation may vary from part to part, which may affect the response of the pressure sensor. Therefore, it was determined that the MG material selected on the plane was appropriate for the model. First, impressions of the maxillary dental model were obtained using alginate impression material (HI-TECHNICOL, GC, Tokyo, Japan), and a hard plaster (ORTHO MAX, JM ORTHO, Tokyo, Japan) was injected to create the plaster dental model. The MG material on the underside of the pressure sensor was pressed against the model. Subsequently, the pressure sensor and circular PMMA plate were fixed to the palatal surface of the maxillary left central incisor, and the MG material on the upper surface of the pressure sensor was pressure welded over it (Figure 2b). The MG-type sensor device was attached to a dental model, and currents were measured at various pressures on the palatal surface of the maxillary left central incisor palate. Each examination was conducted three times and evaluated.

### 2.2. Pressure Measurement with MG-Type Sensor Device

#### 2.2.1. Fabrication of MG-Type Sensor Device

The pressure signal was measured using a Bluetooth low-energy (BLE) communication measurement device (size: 8.5 × 28 × 3.7 mm, sampling interval: 200 ms–30 s, weight: 0.92 g, radio frequency: 2.4 GHz, applied potential: 0–2.048 V, current consumption: 3.5 mA with BLE communication and 2.2 mA without BLE communication, analogue to digital converter resolution: 22 bits, Discretek, Shizuoka, Japan), which had a commercial BLE module (BYSGJNAWY-WX, size: 8.5 × 28 × 3.7 mm, TAIYO YUDEN Co., Tokyo, Japan). A block diagram of the device is shown in Figure S2. The BLE communication measurement device was worn in the oral cavity, and the communication range was measured to be 2 m (Figure S3b). The PC software included functions to search for the periphery of the wireless measuring device for MG-type sensors, receive data, graph data, save data, set data acquisition intervals, and record events. A silver oxide button battery (size: Φ7.9 × 1.65 mm, voltage: 1.55 V, and nominal capacity: 21 mAh, SR716SW, Panasonic, Tokyo, Japan) was used as the power supply for the BLE device. The BLE device was placed on the buccal side of the molar to reduce discomfort while wearing the device. The pressure sensor, circular PMMA plate, and BLE device were placed on the MG material on the lower surface of the pressure sensor. The MG material on the upper surface of the pressure sensor was softened and pressure welded. Subsequently, the MG material was trimmed and thermally welded over the entire circumference using a hot air gun to seal and waterproof it (Figure 2b).

#### 2.2.2. Evaluation of Sensor Characteristics

The fabricated MG device was attached to a dental model, and its response to pressure was examined. In addition, continuous measurements at 1 s intervals were performed via wireless communication to examine the response to various pressures. The examination was conducted three times and evaluated.

### 2.3. Soft Tissue Pressure Measurement in the Mouth

#### 2.3.1. Evaluation of Sensor Devices in the Mouth

Five Japanese male participants were selected for this study (23.5 ± 1.1 y.o.). This study was conducted with the approval of the Ethics Review Committee of the School of Dentistry of Tokyo Medical and Dental University (approval no. D2018-054) in accordance with the latest version of the Declaration of Helsinki. All participants provided their informed consent with an understanding of the purpose and significance of the experiment. The participants were selected based on the following criteria: The participants had permanent dentition, a class I molar relationship, good facial balance determined by visual inspection, and no oral habits. They had no history of orthodontic or surgical treatment, and they were not taking any medications known to affect muscle activity.

First, wireless and wired measurements were performed simultaneously on one participant, and the outputs were compared to examine whether the frequency of the wireless measurement was sufficient. The sensor was placed at the same locations as in the model system described in Section 2.1. The sensor was connected to an extraoral data logger (PicoLog1216, Pico Technology, Cambridge, UK) and a wireless device to compare the wired and wireless outputs produced by the pressure applied from the tongue during swallowing (Figure S4). The sampling interval for the wired measurement was set to 10 ms based on past reports. The sampling interval for wireless measurements was set to 200 ms, the minimum interval for BLE devices.

Tongue pressure during swallowing was measured by swallowing 10 mL water. As previous reports using electropalatography have indicated that the time of contact between the tongue and palate during swallowing is 1.1–2.9 s [35], swallowing was performed every 10 s to ensure that the effect of each swallow on the output was fully eliminated. The measurements were performed 15 times in succession. The participants sat in a chair and fixed their gaze in front with a natural head position, and the measurements were taken. To reduce discomfort with the device and to stabilize the temperature of the sensor, the subjects rested for 5 min after wearing the device before beginning the measurements in subsequent experiments. The water temperature was set to 37 °C to minimize the temperature change during swallowing.

#### 2.3.2. Tongue Pressure Measurement in the Mouth

The fabricated devices were calibrated for each participant before the experiment. The pressure sensor was placed at the same position as in Section 2.3.1. The participants wore the device and performed 10 normal swallows and 10 swallows mimicking tongue thrust-type swallowing, and the output of the device was measured. Tongue thrust swallowing was explained to the participants before the experiment, and they practiced swallowing with their tongue pressed between the maxillary anterior teeth. The accuracy of the simulated behavior was confirmed with visual inspection. The participants were asked to perform a swallow mimicking tongue thrust and to maintain the tongue’s position at the end of the swallow. The tongue apex was then visually confirmed to be positioned between the maxillary anterior teeth. The pressures of the two types of swallow were compared for each participant using a *t*-test. The other experimental conditions were the same as those described in Section 2.3.1.

## 3. Results and Discussion

### 3.1. Suitable Device Structure for Oral Soft Tissue Pressure Measurement

#### 3.1.1. Effect of Diameter of Circular PMMA Plate

**Figure 3:**
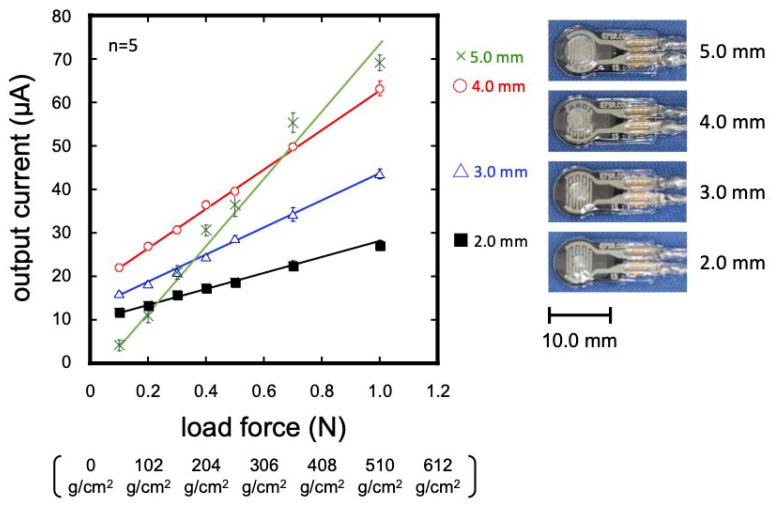
Calibrations with different diameters of PMMA plates.

When the pressure sensor was packed with the MG material, the pressure-sensitive area did not deform uniformly. This leads to a decrease in the sensitivity and reproducibility. The change in the output voltage per unit pressure change is defined as sensitivity. Therefore, a circular PMMA plate with a pressure-transfer element was placed on the pressure sensor for robust sensing. The circular PMMA plates varied in diameter from 2 to 5 mm, and various pressures (51–510 g/cm^2^) were applied for optimization. As shown in Figure 3, a smaller standard deviation was observed when a PMMA plate with a diameter of less than 4 mm was installed. When a PMMA plate with a diameter of 5 mm was installed, it was deemed inappropriate due to the fact of its large sensitivity but large standard deviation. Among the 2–4 mm plates, the largest sensitivity with the minimum standard deviation was observed using a 4 mm diameter PMMA plate. The coefficient of variation (CV) that was calculated using the mean output value and standard deviation was less than 5% over the entire measurement range.

Considering the structure of the resistive pressure sensor, when a PMMA plate with a diameter of 4 mm or smaller was installed, the effective area of the pressure-sensitive part decreased as the PMMA plate became smaller. Consequently, the output decreased (see Figure S5a). As the size of the circular PMMA plate became closer to the size of the sensitive area, the spacer influenced the output (see Figure S5b). The position of the sensor causes variations among sensors, and each sensor needs to be individually calibrated against the pressure. To reduce the variation and the need for the calibration of individual sensors, high-precision positioning may be useful. We would like to introduce quantitative positioning methods in future studies. Based on the above, we determined that the 4 mm diameter PMMA plate was optimal for transmitting the minute pressure generated in the oral soft tissues to the pressure sensor within the MG materials.

#### 3.1.2. Effect of Material on the 2D Sheet-Type Sensor

**Figure 4:**
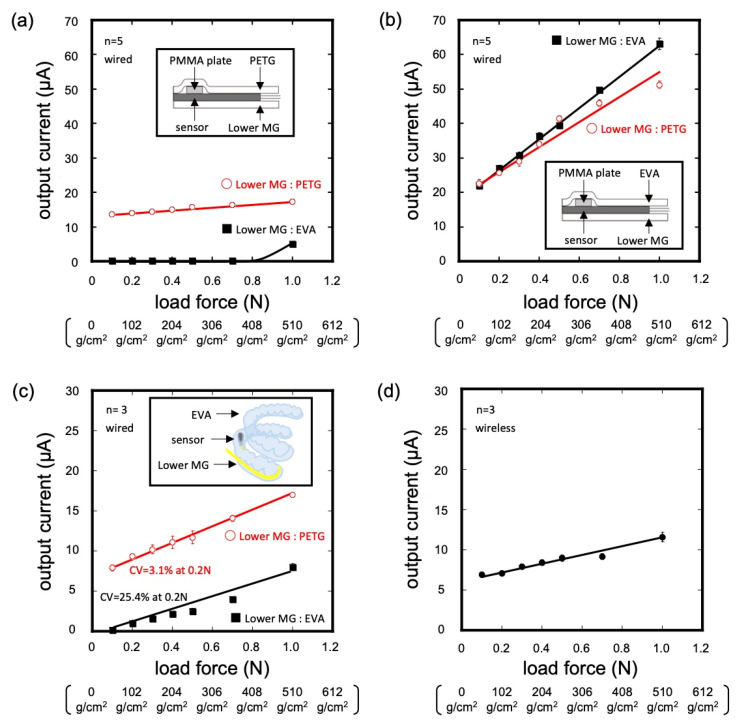
Effect of MG material packing: (**a**) upper MG, PETG (on the plane); (**b**) upper MG, EVA (on the plane); (**c**) upper MG, EVA (on model); (**d**) calibrations on the model of wireless communication.

The effect of each MG material in contact with the upper and lower surfaces of the pressure sensor was evaluated. Figure 4a shows the response when the upper MG material was PETG. With a combination of PETG (lower) and PETG (upper), the pressure sensor was insensitive to a pressure of 51–510 g/cm^2^. In addition, there was no signal at a pressure of 51–357 g/cm^2^ when using the PETG (upper) and EVA (lower) sensors. These properties are unsuitable for oral soft tissue pressure measurements. In contrast, when the upper MG material was EVA, the sensor responded sensitively to pressure, regardless of whether the material on the lower surface was PETG or EVA (Figure 4b). These results were observed because the deformation distance of the MG on the upper surface of the sensor under pressure load decreased owing to the large elastic modulus of the PETG. Based on these results, EVA was used as the MG material for the upper surface of the pressure sensor in the subsequent experiments.

#### 3.1.3. Effect of Material on MG-Type Sensor

The response of a pressure sensor packed in MG material molded three-dimensionally along a dental model was investigated. A pressure sensor and PMMA plate were placed on the palatal surface of the maxillary left central incisor palate. As shown in Figure 4c, the variation at a 102 g/cm^2^ load was large (CV = 25.4%) when the MG material on the lower surface of the pressure sensor was EVA. In contrast, the CV decreased (3.1%) when PETG was used as the MG material for the lower surface. When the lower MG material was EVA, the results were different from those obtained with a flat surface and showed large variation. This may be due to the small gap created between the MG material and the dental model during the three-dimensional formation. When the MG material on the lower part of the pressure sensor was EVA, which has a low elastic modulus, the MG material deformed significantly during pressurization. As a result, there was a large variation in the responsiveness. From the results, we conclude that EVA and PETG are suitable for the fabrication of MG-type sensors as upper and lower MG materials, respectively.

### 3.2. Wireless Measurement

A wireless MG-type pressure sensor device was fabricated by packing a pressure sensor and BLE telemeter connected to a pressure sensor. Sufficient water resistance was confirmed, with no flooding of the device owing to the water immersion. The wireless MG-type sensor showed a stepwise output in response to the applied pressure, similar to that of the wired system (Figure 4d). The output of the wireless measurement was smaller than that of the wired measurement. This was thought to be due to the difference in the properties of the MG material caused by the three-dimensional formation.

### 3.3. Oral Soft Tissue Pressure Measurement

#### 3.3.1. Comparison of Wired and Wireless Systems

**Figure 5:**
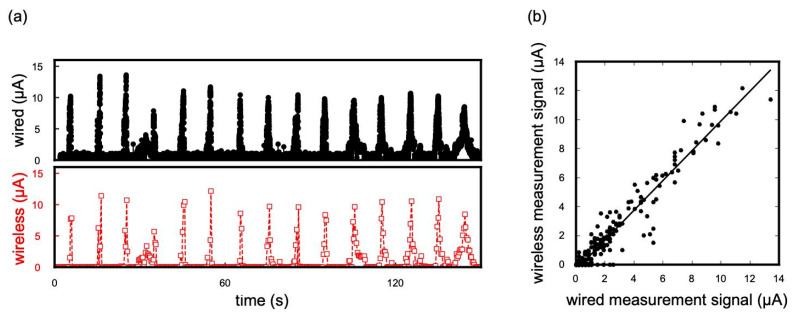
(**a**) Responses from wired and wireless systems; (**b**) relationship between output of wired and wireless measurements.

Tongue pressure against the palatal surface of the maxillary left central incisor during swallowing was measured using wired and wireless systems. In Figure 5a, the upper and lower graphs show the time profiles of the pressure responses obtained using wired and wireless systems, respectively. The number of spikes generated by swallowing was the same in both systems (n = 15). The average relative error, as per Equation (1), for the maximum pressure during a swallowing event measured using the wired and wireless systems was negative 7.5%. This suggests that the data sampling interval of the wireless system was not very problematic in measuring the pressure generated by human swallowing events.

(1) $$\mathrm{Relative}\;\mathrm{error}\;\left(\%\right)=\frac{\mathrm{wireless}\;\mathrm{output}\;\left(\mu A\right)-\;\mathrm{wired}\;\mathrm{output}\;\left(\mu A\right)}{\mathrm{wired}\;\mathrm{output}\;\left(\mu A\right)}$$

The relationship between the wired measurement output and wireless measurement output at each time is shown in Figure 5b. The two measurement methods exhibited high correlation coefficients (R = 0.969). This indicates that the wireless system can monitor not only the maximum pressure but also the change in the output during swallowing. However, although it was short enough to measure tongue pressure during swallowing, a shorter data acquisition interval may be needed if the target of measurement is an event that requires even less time.

#### 3.3.2. Measurement of Tongue Pressure during Swallowing

**Figure 6:**
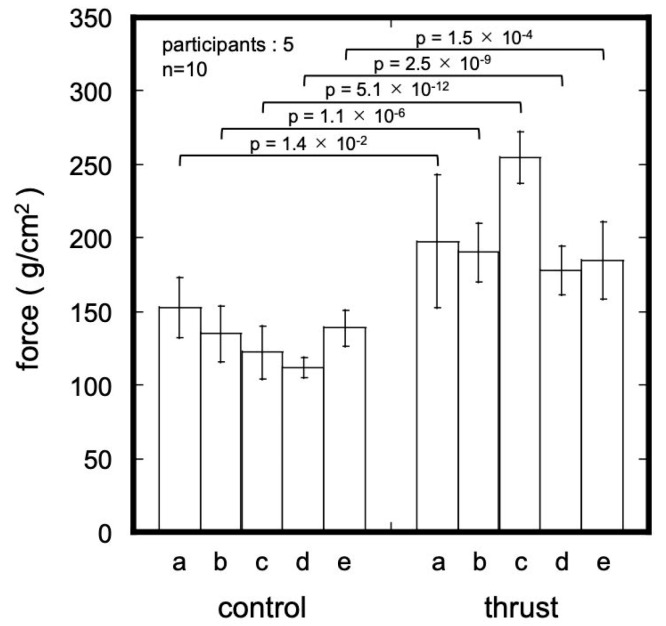
Maximum pressure values for normal swallowing and swallowing while mimicking tongue thrust.

The mean maximum pressure on the palatal surface of the maxillary left central incisor during swallowing is shown in Figure 6. The pressure during swallowing while mimicking tongue thrust was significantly larger than that during normal swallowing in all five participants. The mean and standard deviation of the maximum pressure for the normal swallow in the five participants was 132.14 ± 21.37 g/cm^2^, and the mean of the maximum pressure for swallowing while mimicking tongue thrust-type swallowing was 201.17 ± 38.12 g/cm^2^. This value was significantly higher than that for normal swallowing (*p* = 6.2 × 10^−19^).

Haroon et al. reported that the mean pressure on the palatal surface of the maxillary right central incisor during swallowing in eight males with Angle class I molar relationship was 139.61 ± 1.58 g/cm^2^ [32], which is close to the mean pressure for normal swallowing in the present study. Sinem et al. showed that the tongue pressure on a tongue crib appliance in open bite patients with the tongue thrust significantly decreased, from 216.43 ± 65.79 g/cm^2^ to 142.95 ± 29.2 g/cm^2^, 10 months after the set of the appliance [33]. Note that the tongue crib was an appliance placed just behind the maxillary anterior teeth that improves the tongue thrust habit by bringing the tongue into the correct position. Thus, the results of the study by Sinem suggest that tongue pressure during swallowing can be different with and without tongue thrust in the same participant, which is consistent with the results of the present study. Based on the above results and discussions, it is assumed that the device used in this study can correctly measure tongue pressure.

A comparison of the oral soft tissue pressure measurement devices used in the previous studies is shown in Table 1. Our device is considered to be one of the smallest and most powerful of the few wireless oral soft-tissue pressure measurement devices available. However, this research was conducted under limited conditions. Therefore, it is necessary in future research to show that oral soft tissue pressure can be measured under various conditions, such as during pronunciation, exercise, and sleep. In the future, this device is expected to measure the complex changes in pressure on the teeth over the course of the day.

## 4. Conclusions

In this study, we developed an MG-type device to measure the pressure on teeth from oral soft tissue. A PMMA plate with a diameter of 4 mm was optimal for transmitting the minute pressure generated by the soft tissues in that oral cavity to the pressure sensor in the MG material. EVA and PETG were suitable for fabricating MG-type sensors as materials for the upper and lower MGs, respectively. In addition, a high correlation coefficient (0.969) was observed when comparing wired and wireless devices in human studies. In the measurements of tongue pressure on the maxillary anterior teeth during swallowing, 132.14 ± 21.37 g/cm^2^ for normal and 201.17 ± 38.12 g/cm^2^ for simulated tongue thrust were found to be significantly different using a *t*-test (n = 50, *p* = 6.2 × 10^−19^).

There are also several limitations to this study. First, the study was only validated under the limited condition of swallowing in the sitting position. Future studies should demonstrate that oral soft tissue pressure can be measured under a variety of conditions, including during phonation, exercise, and sleep. Second, the measurement range of the developed device is 51–510 g/cm. This is a sufficient measurement range when the target of measurement is tongue pressure during swallowing. However, in order to measure lip pressure and resting pressure, it is necessary to be able to measure even smaller forces, and the device needs to be improved. Third, the continuous use time is limited due to the battery capacity. Research on wireless power supplies and power harvesting devices could help solve this problem.

This MG-type pressure-sensing device is expected to measure oral soft tissue pressure on teeth, which changes in complex ways throughout the day, under more physiological conditions and over longer periods with further developments.

## Figures and Tables

**Table 1:** Comparison of reported oral soft tissue pressure measurement sensors.

	Wire/ Wireless	Size	Dynamic Range	Sampling Interval	Reference
Takada et al.	Wire	Small	None stated −510 g/cm^2^	5 msec	[19]
Kato et al.	Wireless	Large	None stated −90 g/cm^2^	1.4 msec	[25]
Our device	Wireless	Small	51–510 g/cm^2^	200 msec	This work

## Data Availability

The data that support the findings of this study are available from the corresponding author (K.M.) upon reasonable request.

## Supplementary Materials

The following supporting information can be downloaded at: https://www.mdpi.com/article/10.3390/s23115027/s1, Figure S1: Packing of a pressure sensor and a circular PMMA plate; Figure S2: Algorithms for wireless measurement spacer; Figure S3: Performance of BLE communication measurement device; Figure S4: Protocol for simultaneous wired and wireless measurement; Figure S5: The influence of the pressure sensor.
