# Supplementary material for: Real-Time Continuous Monitoring of Oral Soft Tissue Pressure with a Wireless Mouthguard Device for Assessing Tongue Thrusting Habits
Source: Sensors (Basel). 2023 May 24;23(11):5027. doi: 10.3390/s23115027 (PMC10255842; doi:10.3390/s23115027)
Supplement: Supplementary file 1 [file sensors-23-05027-s001.zip › sensors-2304567-supplementary.pdf]

Supporting Information for

Real-time continuous monitoring of oral soft tissue pressure by wireless mouthguard device for assessing tongue thrusting habit

Hidekazu Matsumoto[1], Keisuke Tomoto[2], Gentaro Kawase[2], Kenta Iitani[2], Koji Toma[2,3], Takahiro Arakawa[2,4], Kohji Mitsubayashi[2,*], and Keiji Moriyama[1]

[1] Department of Maxillofacial Orthognathics, Graduate School of Medical and Dental Sciences, Tokyo Medical and Dental University, Tokyo, Japan
[2] Department of Biomedical Devices and Instrumentation, Institute of Biomaterials and Bioengineering, Tokyo Medical and Dental University, Tokyo, Japan
[3] Department of Electronic Engineering, College of Engineering, Shibaura Institute of Technology, Tokyo, Japan
[4] Department of Electric and Electronic Engineering, Tokyo University of Technology, Tokyo, Japan
* Correspondence: m.bdi@tmd.ac.jp; Tel.: +81-01-5280-8091

Table of contents:

Figure S1. Packing of a pressure sensor and a circular PMMA plate.....……..…...... S-3

Figure S2. Algorithms for wireless measurement spacer...………………..………..S-4

Figure S3. Performance of BLE communication measurement device…......………S-5

Figure S4. Protocol for simultaneous wired and wireless measurement...…..………S-6

Figure S5. The influence of the pressure sensor………………………………..…...... S-7

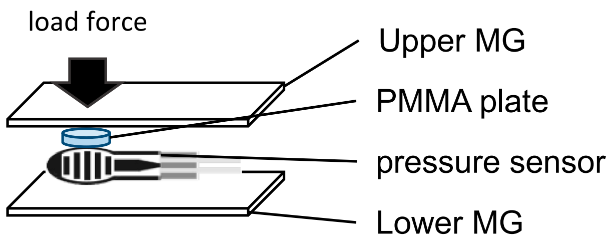

Figure S1. Packing of a pressure sensor and a circular PMMA plate

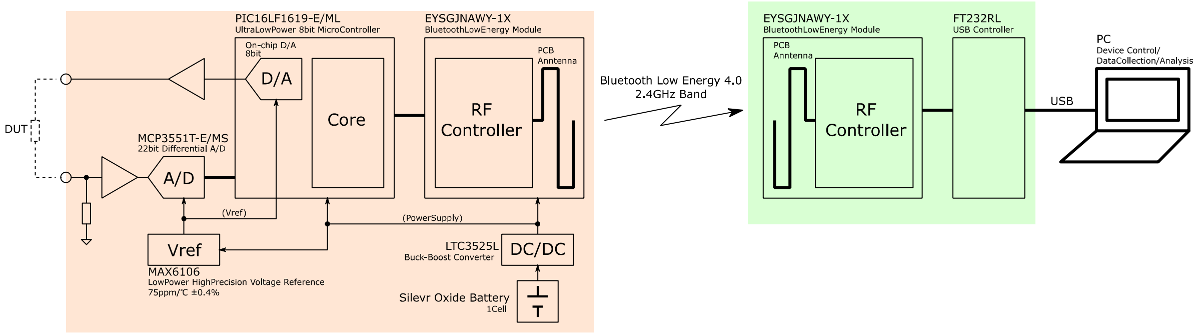

Figure S2. Block diagram for a wireless measurement device

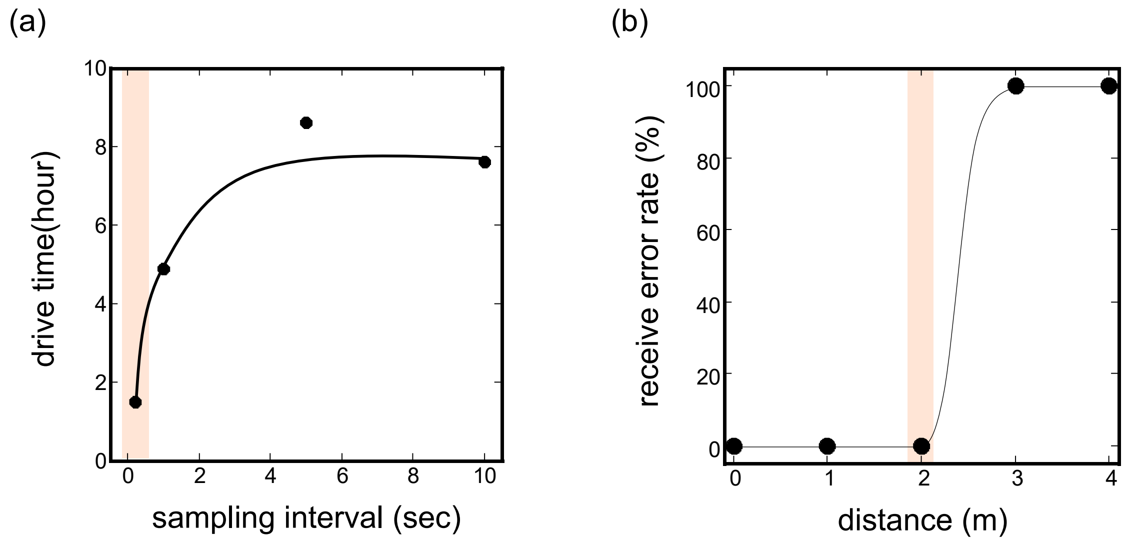

Figure S3. Performance of BLE communication measurement device.

(a)Relationship between sampling interval and drive time, (b) Relationship between communication distance and receive error rate

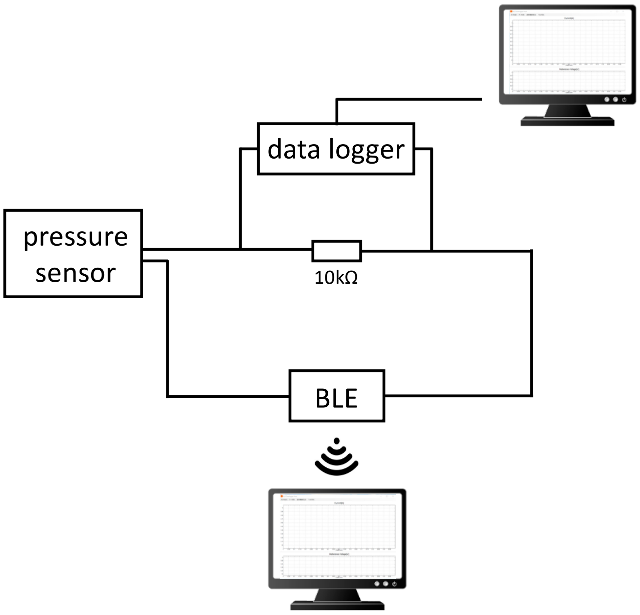

Figure S4. Protocol for simultaneous wired and wireless measurement

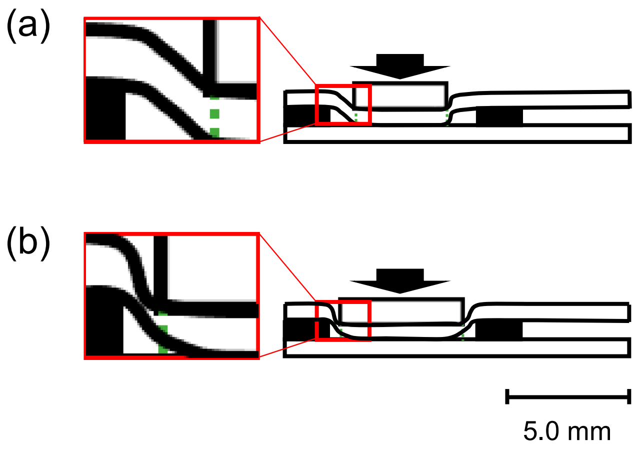

Figure S5. The influence of the pressure sensor spacer (a) the diameter of the circular PMMA plate is 4.0 mm or less, (b) the diameter of the circular PMMA plate is 5.0 mm
